# Trends in hospital admissions due to antidepressant-related adverse drug events from 2001 to 2011 in the U.S.

**DOI:** 10.1186/s12913-017-1993-x

**Published:** 2017-01-19

**Authors:** Harish S. Parihar, Hongjun Yin, Jennifer L. Gooch, Shari Allen, Samuel John, Jianwei Xuan

**Affiliations:** 1 School of Pharmacy, Philadelphia College of Osteopathic Medicine School of Pharmacy-Georgia campus, 625 Old Peachtree Rd NW, Suwanee, GA 30024 USA; 2 Health Economic Research Institute, Sun Yat-sen University, Guangzhou, China

**Keywords:** Adverse drug events, Antidepressants, Hospital admissions, Healthcare Cost and Utilization Project database, Medication errors

## Abstract

**Background:**

Depression is a prevalent mental health disorder and the fourth leading cause of disability in the world as per the World Health Organization. Use of antidepressants can lead to adverse drug events (ADEs), defined as any injury resulting from medication use. This study aimed to examine changes in hospital admissions due to antidepressant-related ADEs (ArADEs) among different socio-demographic groups and changes in lengths of stay (LOS) and hospital charges in ArADE admissions from 2001 to 2011.

**Methods:**

The Healthcare Cost and Utilization Project database was used. ArADE admissions in different socio-demographic groups were examined including characteristics such as age, gender, rural/urban, and income. LOS and hospital charges for ArADE cases were compared between 2001 and 2011. Chi-square test and t test were used for statistical analyses.

**Results:**

There were 17,375 and 20,588 ArADE related admissions in 2001 and 2011, respectively. There was a 17.6% increase among the group of 18 to 64 years old and a 64.8% increase among the group of 65 years or older while the other age groups experienced decreased admission rates. Males and females had similar increases. Patients from the lower income areas experienced a two-fold increase while those from the higher income areas experienced a decrease. The mean LOS for all ArADE related admissions increased from 2.18 to 2.81 days and mean hospital charges increased from $8,456.2 to $21,572.5.

**Conclusions:**

There was an increase in ArADE hospital admissions. The greater increase in ArADE admissions among elderly, urban or low-income patients should be noted and addressed by practitioners and policy makers. The large increase in hospital charges needs further research.

## Background

Depression is a prevalent mental health disorder and the fourth leading cause of disability in the world as per the World Health Organization (WHO) [1]. Antidepressants are the most common treatment options for depression. In addition, antidepressants are also prescribed for other conditions such as dysthymia, bipolar depression, schizoaffective disorder, post-psychotic depression, generalized anxiety disorder, panic disorder, social phobia, substance abuse disorders, anorexia, bulimia, obsessive compulsive disorder, post-traumatic stress disorder, and chronic pain syndromes [2]. The prevalence of antidepressant use has trended upwards in the United States in the last decade. In 2000 about 6.5% of the adult population in the US was treated with antidepressants while 10.4% were prescribed the medications in 2010 [3]. Long-term users of antidepressants increased from 3.0% to 6.9% over the same time period.

The Institute of Medicine defines adverse drug events (ADEs) as any injury resulting from medication use [4]. The American Society of Health-System Pharmacists (ASHP) defines a significant ADR as any unexpected, unintended, undesired, or excessive response to a drug that requires discontinuation of the drug, changing the drug therapy, modifying the dose, necessitates admission to a hospital; prolongs stay in a health care facility, necessitates supportive treatment, significantly complicates diagnosis, negatively affects prognosis or results in temporary or permanent harm, disability, or death [5]. Severe adverse drug effects caused by antidepressants may lead to mortality, including self-inflicted injuries, myocardial infarction, stroke/transient ischemic attack, falls, fractures, upper gastrointestinal bleeding, epilepsy/seizures, and others [6]. In 2011, 89,000 emergency room visits in the U.S were due to adverse drug events (ADEs) related to psychiatric medications including antidepressants [7]. Although, not all ADEs are preventable, there is data to support that approximately one-quarter of all harmful ADEs are preventable [8]. Bates and colleagues found that antidepressants are one of three classes of medications that are significantly associated with preventable ADEs among hospitalized patients [9].

The prevalence and incidence of ADEs may be related to socio-demographic factors. Previous studies showed that among the causes of emergency department visits or hospitalizations, patients aged 65 years or older were associated with higher rate of ADEs than younger patients [10]. A review by Tache et al on prevalence of ADEs among ambulatory care patients showed that elderly patients had higher prevalence rate than younger adult patients, who had higher prevalence rate than pediatrics patients [11]. Studies on the relationship between other socio-demographic factors and ADEs are scarce in the literature. This study aimed to examine changes in incidence of hospital admissions due to antidepressant-related ADEs (ArADEs) from 2001 to 2011 in the U.S., whether the changes are similar across different age groups, if other socio-demographic factors (sex, income, and urban/rural setting) influence any of the observed changes in different age groups; and to examine changes in lengths of stay (LOS) and hospital charges in ArADE hospitalizations from 2001 to 2011.

## Methods

A retrospective secondary data analysis was conducted using Healthcare Cost and Utilization Project (HCUP) National Inpatient Sample (NIS) database. This study was approved by The Institutional Review Board of the Philadelphia College of Osteopathic Medicine.

The HCUP is a family of databases sponsored by the Agency for Health Research and Quality and covers community hospitals from more than 40 states [12]. HCUP databases are derived from hospital administrative data, which include diagnoses and procedures, discharge status, patient demographics, and charges as well as characteristics of the hospitals such as teaching status, urban or rural location and size. We used the National Inpatient Sample (NIS) data from the HCUP database. The NIS data included inpatient care from all-payer hospitals. After applying weights, the NIS data estimate about 36 million hospitalizations nationally [13].

In the HCUP data, age was recorded in years and race was categorized into six groups: white, black, Hispanic, Asian and Pacific Islander, Native American, and other. Location of a hospital was recorded as either rural or urban. Median income of zip code of patient’s residence was categorized into four quartiles.

### Identification of ArADE admissions

ADEs are defined as any injury resulting from medication use. For this study, Illicit drug use and cases of intentional harm or self-inflicted injury were excluded (ICD codes E950.0-E950.9, Suicide and self-inflicted poisoning; E962.0-E962.9, Assault by poisoning; E980.0-E980.9 Poisoning undetermined whether accidentally or purposely inflicted). The HCUP database recorded up to 15 diagnoses in 2001 and up to 25 diagnoses in 2011 for general ADE-associated diagnosis of each inpatient stay record. The diagnoses were recorded in International Statistical Classification of Diseases and Related Health Problems, 9th revision (ICD-9) codes. The ICD-9 codes used to identify ArADEs were retrieved from a report by Lucado et al. [14]. Primary diagnoses of ArADE were considered as an indicator of ArADE admissions.

### Statistical analysis

Weights of individual inpatient stays provided in the database were used to estimate the national total. ArADEs in different socio-demographic groups were examined including age, race, gender, rural/urban, and median income in a patient’s residential area. Age was categorized into 0 to 6, 7 to 17, 18 to 64, and 65 years or older. LOS and hospital charges for ArADE cases were compared between 2001 and 2011. Chi-square test was used to compare changes in ADEs relative to the change in total number of hospital admissions. Student’s T-test was used to compare changes in LOS and hospital charges. Given the highly skewed distribution of LOS and hospital charges, the two variables were transformed by taking square root before T-tests were conducted. A p-value of ≤0.01 was considered statistically significant. To more accurately reflect the changes in each subgroup relative to the change in total ArADE admissions over time, percentage changes in rates (rate was calculated as ArADE admission in a subgroup divided by total ArADE admissions in the corresponding year) for each subgroup were also calculated (Table 1). To simplify the presentation for changes by age and income, patients from areas with income in the lower two quartiles were lumped together (“bottom-half”) and patients from areas with income in the upper two quartiles were lumped together (“top-half”) (Table 3).

**Table 1:** Changes in ArADE admissions by age

	2001	2011	Percentage Change in admissions rate^a^
N (all admissions)	37,185,482	38,560,751	3.7^b^
ArADE admissions*	17,375	20,588	15.32
Age (years)^†^			
≤ 6	272	233	-25.72
7–17	1,863.4	1,586	-26.20
18–64	14,280	16,787	1.94
≥ 65	656.37	1,082	42.94

^a^Percentage changes in rate. Rate was calculated by dividing ArADE admissions or admissions in each age group by total number of all admissions of the respective year. Percentage change was calculated using 2001 rate as the base

^b^Rate change in number of admissions from 2001 to 2011 using 2001 as the base

*p ≤ 0.01, which indicates there is significant difference between the change in ArADE admissions and the change in overall hospital admissions in the U.S

^†^ *p* ≤ 0.01, which indicates changes between different age groups are significantly different

Note: The numbers by age groups were counted after excluding records with missing data. Hence the sum of different age groups is less than the total number ArADE admissions

## Results

Changes in number of ArADE admissions from 2001 to 2011 between different demographic groups are presented in Tables 1, 2, 3 and 4. Due to a large number of records in 2001 that were missing race data, results on changes of ArADE admissions by race are not presented. There were 17,375 and 20,588 ArADE admissions in 2001 and 2011 respectively, this increase was statistically significant (*p* < 0.01, Table 1). After excluding records with missing data in age, sex, median income in a patient’s residential zip code, or hospital setting (urban/rural), the numbers were 17,072 and 19,688 for 2001 and 2011 respectively and the result was still statistically significant. The four age groups experienced different changes in ArADE admissions (*p* < 0.01, Table 1). Specifically, ArADE admissions in ≤6 years and 7–17 years old patients decreased 14.3% and 14.9% respectively. Conversely, the admission in the group of 18 to 64 years and ≥65 years showed an increase of 17.6% and 64.8% respectively. Rates for ≤6 years and 7–17 years old patients decreased by 25.72% and 26.20% respectively. Conversely, rates for the groups of 18–64 years and ≥65 years showed an increase of 1.94% and 42.94% respectively.

**Table 2:** Changes in ArADE Admissions from 2001 to 2011 by age and sex

	2001		2011		Percentage change in admissions rate^a^	
	Females	Males	Females	Males	Females	Males
ArADE admissions	11,342	5,730	12,854	6,834	13.3^b^	19.3^b^
ArADE admissions by age group						
≤ 6	150	122	130	103	-23.5	-29.2
7–17	1,325	538	1,155	430	-23.1	-33.0
18–64*	9,410	4,871	10,800	5,988	1.3	3.1
≥ 65	457	199	769	313	48.5	31.9

^a^Percentage changes in rate. Rate was calculated by dividing ArADE admissions in the corresponding subgroup by the corresponding column total of ArADE admissions of the respective year. Percentage change was calculated using 2001 rate as the base

^b^Rate change in number of ArADE admissions from 2001 to 2011 using 2001 as the base

*p ≤ 0.01, which indicates change in rates over time were significantly different between males and females in the specific age group

**Table 3:** Changes in ArADE Admissions from 2001 to 2011 by age and neighborhood income

	2001 (income)		2011 (income)		Percentage change in admissions rate^a^	
	Bottom 50%	Top 50%	Bottom 50%	Top 50%	Bottom 50%	Top 50%
ArADE admissions*	4,972	12,100	10,624	9,054	113.7^b^	-25.2^b^
ArADE admissions by age group						
≤ 6	109	163	117	115	-49.8	-5.7
7–17^†^	509	1,354	820	766	-24.6	-24.4
18–64^†^	4,150	10,131	9,233	7,545	4.1	-0.5
≥ 65^†^	204	452	454	628	4.2	85.7

^a^Percentage changes in rate. Rate was calculated by dividing ArADE admissions in the corresponding subgroup by the corresponding column total of ArADE admissions of the respective year. Percentage change was calculated using 2001 rate as the base

^b^Rate change in number of ArADE admissions from 2001 to 2011 using 2001 as the base

*p ≤ 0.01, which indicates there is a significant difference between areas of bottom-half income and top-half income with regard to change in ArADE admissions

^†^ *p* ≤ 0.01, which indicates change in rates over time were significantly different between areas of bottom-half income and top-half income in the specific age group

**Table 4:** Changes in ArADE Admissions from 2001 to 2011 by age and urban/rural setting of a hospital

	2001		2011		Percentage change in admissions rate^a^	
	Urban	Rural	Urban	Rural	Urban	Rural
ArADE admissions*	13,677	3,395	16,789	2,899	22.8^b^	-14.6^b^
ArADE admissions by age group						
≤ 6^†^	190	82	223	10	-4.4	-85.8
7–17^†^	1,499	364	1,378	208	-25.1	-33.1
18–64^†^	11,443	2,838	14,214	2,573	1.2	6.2
≥ 65^†^	545	111	974	108	45.6	13.9

^a^Percentage changes in rate. Rate was calculated by dividing ArADE admissions in the corresponding subgroup by the corresponding column total of ArADE admissions of the respective year. Percentage change was calculated using 2001 rate as the base

^b^Rate change in number of ArADE admissions from 2001 to 2011 using 2001 as the base

*p ≤ 0.01, which indicates there is a significant difference between urban and rural areas with regard to change in ArADE admissions

^†^ *p* ≤ 0.01, which indicates change in rates over time were significantly different between areas of bottom-half income and top-half income in the specific age group

There was no significant difference between males and females in terms of change over time in overall ArADE admissions (*p* > 0.01, Table 2). When specific age groups were examined, there was a significantly larger increase in rate of ArADE admissions among males than their female counterparts for the group of 18 to 64 years old (*p* < 0.01). Specifically, ArADE admissions in female and male patients increased by 14.8% and 22.9% respectively. Changes in rates in female and male patients were 1.3% and 3.1% respectively. There was no significant difference found between males and females for the other three groups (≤6, 7–17, and ≥65 years old) in terms of change (*p* > 0.01).

As shown in Table 3, there was a significant larger increase in ArADE admissions among patients from areas of bottom-half income than their top-half counterparts (*p* < 0.01). Specifically, ArADE admissions in bottom-half areas increased by 113.7% while ArADE admissions in top-half areas decreased by 25.2% respectively. Changes over time were significantly different between the bottom-half areas and top-half areas for the groups of 7 to 17 years old, 18 to 64 years old and ≥65 years old (*p* < 0.01). For the group of 7 to 17 years old, ArADE admissions changed by 61.6% and -43.4% in the areas of bottom-half income and areas of top-half income respectively. For the group of 18 to 64 years old, ArADE admissions changed by 122.5% and -25.5% in the areas of bottom-half income and areas of top-half income respectively. For the group of ≥65 years old, ArADE admissions changed by 122.5% and 38.9% in the areas of bottom-half income and areas of top-half income respectively. However, the changes in rates in the corresponding income groups were much greater for the ≤6 and ≥65 years old groups than the 7 to 17 and 18 to 64 years old groups. Specifically, for the ≤6 years old group, changes in rates in bottom-half and top-half areas were -49.8% and -5.7% respectively. For the ≥65 years old group, changes in rates in the bottom-half and top-half areas were 4.2% and 85.7% respectively.

The changes in ArADE admissions from 2001 to 2011 by age and urban/rural setting of a hospital are presented in Table 4. There was a 22.8% increase in ArADE admissions among urban hospitals and a 14.6% decrease among rural hospitals. This change was statistically significant (*p* < 0.01). Changes over time were significantly different between the urban and rural hospitals for all age groups as presented in Table 4 (*p* < 0.01). The changes in rates among urban and rural hospitals showed similar direction (increase or decrease) for each age group.

As shown in Table 5, the mean LOS of ArADE admissions were 2.18 days in 2001. Change in mean LOS of ArADE admissions from 2001 to 2011 was 0.63 days, which was statistically significant (*p* < 0.01). Changes in mean LOS of ArADE admissions for the 7–17 years old and 18–64 years old groups were statistically significant, which was 0.42 and 0.64 days respectively.

**Table 5:** Length of stay (LOS) of ArADE admissions in 2001 and 2011

	Mean (SD, Median)		Change in mean LOS
	2001	2011	
LOS of all ArADE admissions	2.18 (6.31, 1)	2.81 (7.74, 2)	0.63*
LOS by age group			
≤ 6	0.89 (1.30, 1)	1.12 (1.78, 1)	0.23
7–17	1.46 (2.71, 1)	1.88 (5.38, 1)	0.42*
18–64	2.20 (6.40, 1)	2.84 (7.74, 2)	0.64*
≥ 65	4.17 (9.59, 2)	3.97 (10.16, 2)	-0.20

*p < 0.01

Mean hospital charges more than doubled from $8,456.2 to $21,572.5 (*p* < 0.01, Table 6) for ArADE admissions when all age groups were considered. When the different age groups were analyzed separately, changes in mean hospital charges were statistically significant for all four age groups (*p* < 0.01).

**Table 6:** Hospital Charge for ArADE admissions in 2001 and 2011

	Mean (SD, median)		Change in mean hospital charge
	2001($)	2011($)	
Hospital charge of all ArADE admissions (SD, median)	8,456.2 (34,996.2, 5,022.0)	21,572.5 (94,128.3, 11,899.0)	13,116.3*
Hospital charge by age group (SD, median)			
≤ 6	2,535.0 (3,116.3, 1,354.4)	6509.8 (11,256.9, 3,594.8)	3,974.8*
7–17	4,931.8 (11,650.1, 2,666.0)	11,975.7 (43,338.3, 6,656.6)	7,043.9*
18–64	8,681.3 (35,751.9, 5,111.5)	22,151.5 (96,347.3, 11,909.1)	13,470.2*
≥ 65	16,038.4 (55,787.2, 9,078.9)	29,990.7 (115,441.0, 14,979.1)	13,952.3*

*p < 0.01

## Discussion

The present study was the first to examine the change in ArADE hospital admissions over time (between 2001 and 2011) in the U.S. First, there was a significantly higher increase in ArADE admissions as compared to all-cause admissions. According to data published by Agency for Healthcare Research and Quality and IMS Health, the number of prescriptions for antidepressants increased by approximately 98% from 2001 to 2011 [15, 16]. Thus, the percentage increase in ArADE admissions is less than the percentage increase in prescriptions. However, the greater increase in ArADE admissions than all-cause admissions suggests that ArADE has become a bigger social and economic burden in the U.S. Other factors that may explain the overall increase in ArADE admissions include change in antidepressants most widely prescribed and the introduction of new antidepressants onto the market [17–19].

This study found that the overall increase in ArADE admissions is mainly attributed to increases among adult patients, since patients 17 or younger exhibited decrease in ArADE admissions. The group of 65 or older experienced the greatest increase in both absolute numbers and relative proportions to the total ArADE admissions (rates). According to a study by Olfson and Marcus [18], the rate of antidepressant use over time has had similar increase across all age groups. Increase in the U.S. population from 2000 to 2010 has been 2.6%, 11.6% and 15.1% for < =17, 18 to 64, and > =65 age groups respectively [20]. Thus, the change in size of population alone cannot explain the different trends in ArADE admissions. The greatest increase in ArADE admissions among patients 65 or older may be due to increased number of prescriptions, drug-drug interactions because of polypharmacy, weaker physical condition than younger patients, and comorbidities. More investigation on the potential causes of these different trends is needed.

In addition to unequal changes between age groups, the change in ArADE admissions from 2001 to 2011 was also unequal between sexes, areas of different income levels and urban-rural settings. Olfson and Marcus found that male patients diagnosed with depression had a greater increase in prescriptions per person [20], which may partially explain our finding that male patients had a higher increase in ArADE admissions within the 10 years time period. A closer look at the data showed that the higher increase among males than females occurred in patients 18–64 years old.

When all age groups were considered, areas of the highest income quartile experienced a decrease in ArADE admissions while areas of the lowest income quartile experienced almost a five-fold increase (quartile results were not presented in the tables for simplification purpose). Among patients 65 years or older, areas of the highest income quartile experienced the least increase while the lowest income quartile experienced almost a six-fold increase. This is contrary to the findings by Zhang and colleagues [21] who found that socio-economic disadvantage is not associated with a higher risk of adverse drug reactions. The different trends in areas with different income levels observed in the present study may be partially explained by what was found by Lorant and colleagues [22]. In a meta-analysis, Lorant and colleagues reported that individuals with lower socio-economic status (SES) had higher risk of new depression episodes than those with higher SES and the difference in risk of persisting depression between low and high SES individuals was even greater. However, such difference in risk of depression cannot completely explain the different trends over time found in the present study. Other potential factors may include poor quality of care to patients with depression and less access to timely care in lower-income patients, which could lead to greater increase in ArADE admissions.

The greater increase in ArADE admissions among urban patients in comparison to rural patients can be partially explained by the greater increase in urban population. According to the U.S. census bureau [23], urban population grew by 12.1% while rural population grew only 0.3% from 2000 to 2010. However, the magnitude of increase in ArADE admissions among urban patients is much greater than the population growth.

Overall, both mean LOS and mean hospital charge for ArADE admissions from 2001 to 2011 showed an increase from 2001 to 2011. Combining with the increased number of ArADE admissions, this suggests that ArADE incurred an increased burden to the society. This study also showed that different age groups experienced different magnitudes of increase in LOS and hospital charges over the time period. While LOS of ArADE admissions increased only slightly when all age groups were considered together, the average and median hospital charges more than doubled. This can partially be attributed to medical cost inflation, which rose by 47.1% in the same time period [24]. The additional increase in hospital charges may be due to the change in case mix associated with greater share of elderly patients and patients who did not receive timely care among other factors. As shown in the results, mean LOS for patients 65 or older actually decreased (although insignificant) while mean hospital charge for that group increased significantly.

The present study has several limitations. In this study, it was assumed that diagnosis recorded in the first ICD-9 code of a hospital admission is the primary diagnosis that caused the admission. Instances of illicit drug use and cases of intentional harm or self-inflicted injury were identified based on ICD code and excluded from the study.. However, we acknowledge that ICD codes are optional for billing purposes and notoriously non-sensitive when used for ADE outcomes of interest. Especially in the case of self-assault or attempted suicide, clinicians may be wary to document such information due to legal and social implications or simply due to lack of background information on the patients. Thus, although some cases of intentional self-harm were excluded from the analyses; however there is a possibility of misclassification bias. We were also limited by the information available in the HCUP databases. Several states do not report the race variable. Hence the incidence of ArADE admissions and differences between different racial groups couldn’t be compared. Additionally, individual patients cannot be followed on different admissions to a hospital due to the lack of a common ID for the same patient. As a result, a patients with multiple ArADE admissions to the included hospitals was counted as multiple patients. Also, it would be noteworthy to mention that there has been a great deal of awareness and research regarding ADEs in general since early 2000. Provider awareness to more appropriately diagnose and code these related conditions (for treatment and billing purposes) could therefore account for the perceived ‘higher’ incidence in 2011.

## Conclusion

ArADE hospital admissions had significant increase from 2001 to 2011 in the United States especially in the elderly, areas of lower-income and urban patients. The increases have incurred a greater economic burden to the society. Further research is needed to investigate the root causes of these increases and the large increase in hospital charges. It would also be beneficial to examine whether similar trends among the different socio-demographic groups exists for hospital admissions related to ADEs of other drug classes. Currently, various CDC and FDA led programs are being executed with the goal of increasing awareness to medication safety and reducing ADEs. Probably, actions to bolster these initiatives and implementation of novel public health actions are needed to address the increase in ArADE admissions.

## Abbreviations

ADEs: Adverse drug events
ArADEs: Antidepressant-related ADEs
LOS: Lengths of stay
WHO: World Health Organization
ASHP: The American Society of Health-System Pharmacists
HCUP: Healthcare Cost and Utilization Project
NIS: National Inpatient Sample
ICD-9: International Statistical Classification of Diseases, 9th revision
